# Exogenous acetate mitigates later enhanced allergic airway inflammation in a menopausal mouse model

**DOI:** 10.3389/fcimb.2025.1543822

**Published:** 2025-04-10

**Authors:** Evelyn Roxana Perez Umana, Eduardo Mendes, Mateus Campos Casaro, Mariana Lazarini, Fernando A. Oliveira, Anne I. Sperling, Caroline Marcantonio Ferreira

**Affiliations:** ^1^ Institute of Environmental, Chemistry and Pharmaceutical Sciences, Department of Pharmaceutics Sciences, University Federal de São Paulo, Diadema, Brazil; ^2^ Cellular and Molecular Neurobiology Laboratory (LaNeC), Center of Mathematics, Computing and Cognition (CMCC), Federal University of ABC, São Bernando do Campo, Brazil; ^3^ Pulmonary and Critical Care Laboratory, Department of Medicine, University of Virginia, Charlottesville, VA, United States

**Keywords:** acetate, allergic airway inflammation, menopause, regulatory T cells, gut-lung axis

## Abstract

**Introduction:**

Asthma, an inflammatory lung disease, disproportionately affects women in adulthood and is associated with a decline in estrogen levels during the menstrual cycle and menopause. To study asthma symptoms during menopause, we used a mouse model of postmenopausal asthma via ovariectomy (OVx). Similar to human menopause, we previously discovered that re-exposure of allergic OVx mice to allergen exacerbates lung inflammation. Surprisingly, we found that probiotic treatment alleviates this inflammatory exacerbation and produces acetate as one of its metabolites. Here, we investigate whether exogenous acetate alone can inhibit the exacerbation of experimental asthma in menopause.

**Methods:**

Mice received acetate administration before and during sensitization. After challenge and OVx the mice were subjected to a second challenge to test whether acetate protected against airway inflammation after menopause induction.

**Results:**

Acetate administration reduced all lung T2 inflammatory responses, as well as the serum immunoglobulin (IgE) level. Early acetate treatment led to an increase in regulatory T cells, even 3 weeks after cessation of the treatment, suggesting that the increase in Treg percentage is associated with the reduction of type 2 inflammation in the airways after menopause induction, indicating its potential role in this process. Given the significant role of the lung-gut axis in asthma and the association of asthma and menopause with intestinal dysfunctions, this finding is particularly relevant; we also analyzed several markers of intestinal integrity. Compared with sham-operated mice, rechallenged allergic menopausal mice had a reduction in the intestinal epithelial genes, MUC2 and OCLN, and preventive supplementation with acetate returned their expression to normal. No change was found in menopausal mice without allergic inflammation.

**Conclusion:**

In conclusion, treatment with acetate prior to estrogen level decline protects sensitized and challenged mice against later airway T2 inflammation and may restore gut homeostasis.

## 1 Introduction

Millions of women will experience menopause, a life phase often associated with increased risk factors and declines in various bodily functions, which can, in turn, promote disease later in life (McCarthy and Raval, 2020; Peacock et al., 2023). For example, asthma is a chronic inflammatory airway disease that affects more than 300 million people, and the incidence and severity of asthma are greater in females than in males, particularly between the 4th and 6th decades of life. In women older than 50 years of age, menopause may coincide with the onset of asthma, or in females with a history of asthma, menopause may be associated with the worsening of symptoms (Colombo et al., 2019; Chowdhury et al., 2021). These findings suggest that female sex hormones are involved in the development of the disease and that their decline and fluctuation affect asthma (Fish, 2008; Prudente De Carvalho Baldaçara and Ii, 2017). The interaction between estrogen and the immune system is complex, and much is still unknown about how to modulate this interaction to alleviate symptoms or prevent asthma. Here, we focus on preexisting asthma that is exacerbated during the menopausal phase.

Animal models have been used to understand menopausal issues and consequently to improve quality of life. Since asthmatic women in either the perimenstrual or perimenopausal stages may experience worsening asthma symptoms, we developed a model to investigate the role of female sex hormones in the modulation of experimental allergic lung inflammation (Mendes et al., 2017). The mice were sensitized and challenged ten days before ovariectomy (OVx) and rechallenged ten days after OVx. In this model, we mimicked the worsening of asthma during menopause. Considering the importance of the intestinal microbiota for immunological responses in the respiratory system, known as the gut−;lung axis, mice were supplemented with a probiotic, Bifidobacterium longum 51A, that prevented the exacerbation of airway inflammation in allergic OVx mice (Mendes et al., 2017).

We have identified that *Bifidobacterium longum* 5^1A^ produces acetate as one of its metabolites (Mendes et al., 2017). Acetate treatment has been investigated in various inflammatory diseases, including experimental asthma and gut inflammatory disorders, and it has shown anti-inflammatory effects (Thorburn et al., 2015; Moffett et al., 2020; Shen et al., 2021; Zhang et al., 2021; Hosmer et al., 2023). However, its role in postmenopausal asthma remains unknown. Here, we test the hypothesis that exogenous acetate, a short-chain fatty acid, contributes to reducing menopausal asthma exacerbation by administering it to the mice. Considering the importance of the gut-lung axis in this model and the association between respiratory diseases, including asthma, and intestinal disorders, we also investigated how acetate affects gut-specific gene expression and mucus production in our model of menopausal asthma. Furthermore, since menopausal women are more prone to experiencing intestinal imbalances (Chen and Madak-Erdogan, 2018; Peters et al., 2022), we explored whether acetate could influence these aspects as well.

Considering that, the gut−;lung axis is important in this model, and respiratory diseases, including asthma, are associated with intestinal disorders. Furthermore, menopausal women are more prone to experiencing intestinal imbalances (Chen and Madak-Erdogan, 2018; Peters et al., 2022), thus we explored the effects of acetate on gut-specific gene expression and mucus production in our model of menopausal asthma.

## 2 Materials and methods

### 2.1 Study design

**Figure 1:**
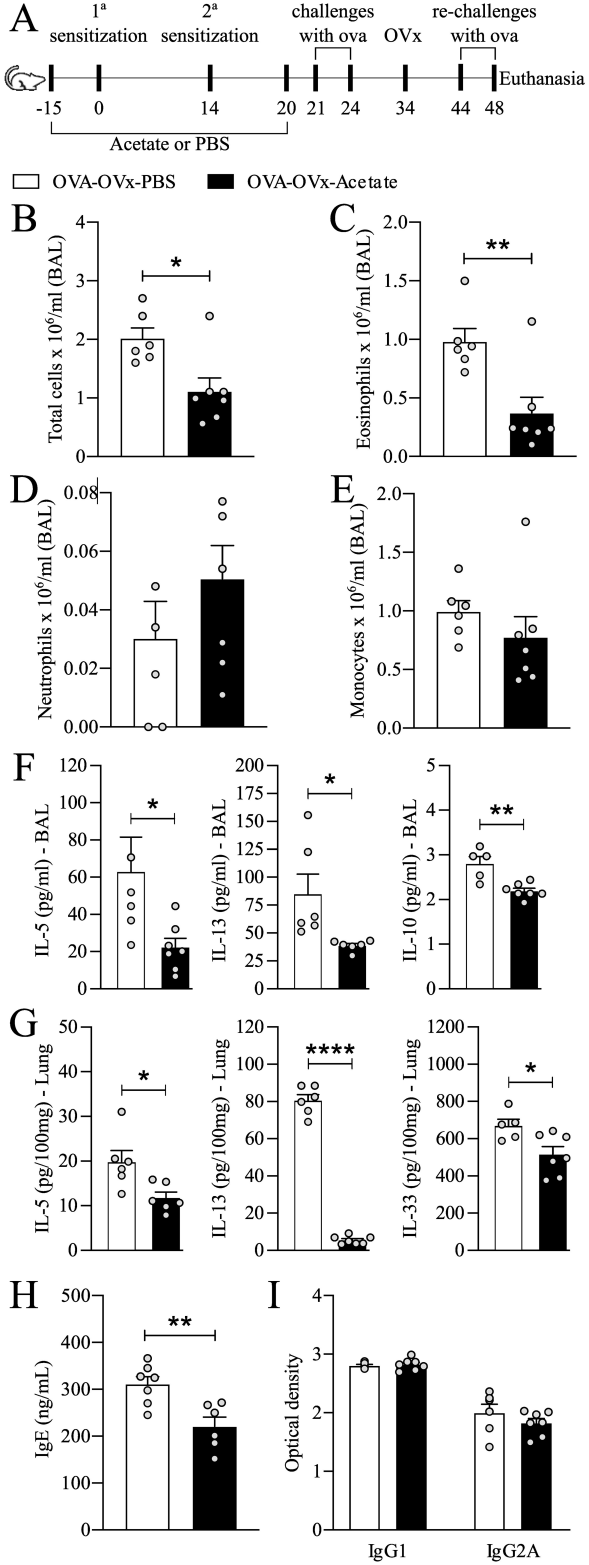
Preventive acetate treatment reduces allergic airway inflammation in re-challenged OVx allergic mice. **(A)** Acetate was administered 15 days prior to sensitization and continued until one day before the first challenge (day 20).Ten days after the last challenge, the ovaries were removed. Ten days after ovariectomy (OVx), animals underwent rechallenge. All parameters were assessed 24 hours after the last rechallenge. **(B)** Quantification of the total number of cells, **(C)** eosinophils, **(D)** neutrophils, and **(E)** mononuclear cells in the BAL fluid. **(F)** Concentrations of IL-5 in lung tissue and **(G)** concentrations of IL-5, IL-10, and IL-13 in BAL fluid. **(H)** IgE concentration and **(I)** IgG and IgG2A concentrations in serum. All the results represent data obtained from two separate experiments and are presented as the means ± SEMs (n = 6–7 in all the groups). Statistical significance was determined via Student’s t test. *P < 0.05, **P < 0.01, **** means P < 0.0001.

**Figure 2:**
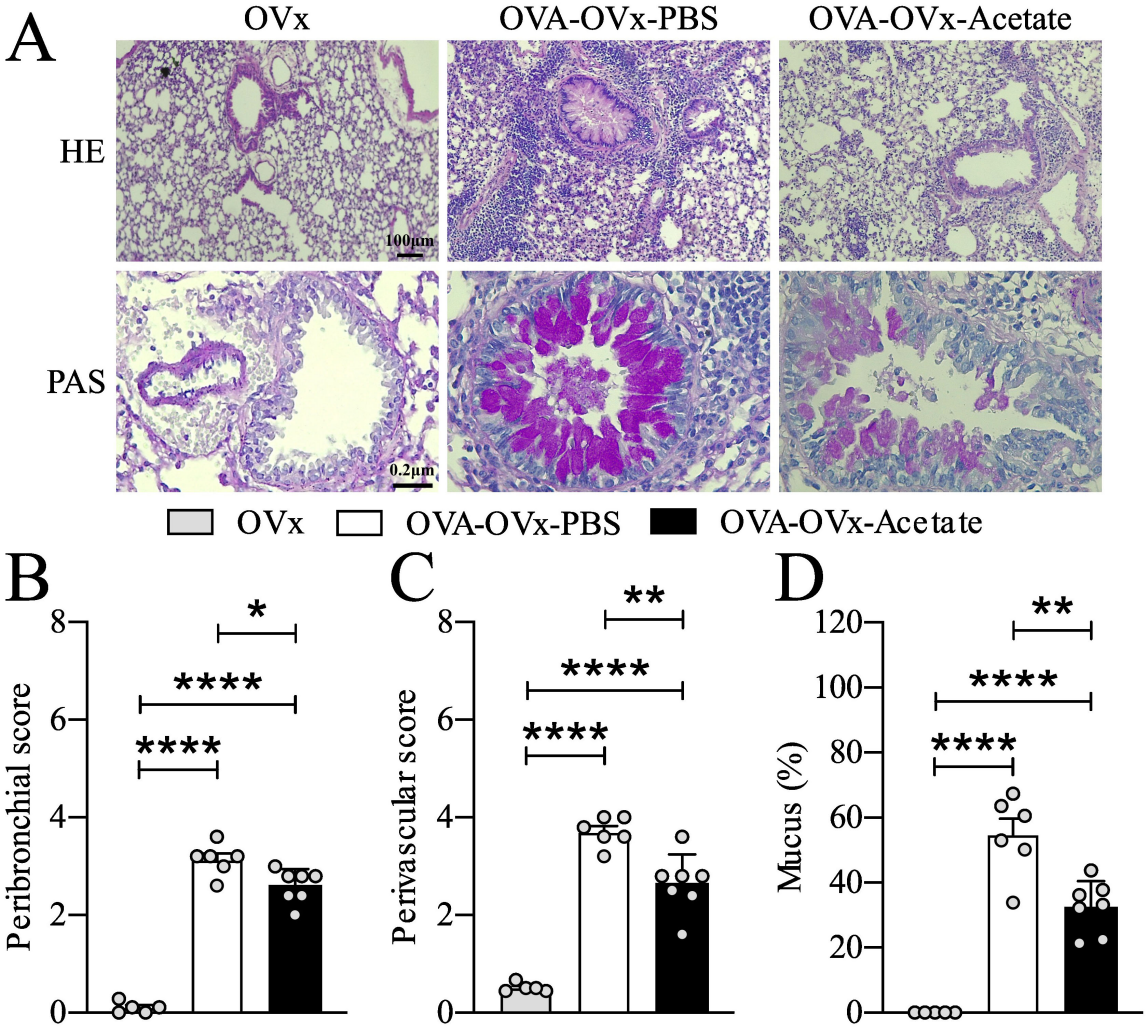
Preventive acetate treatment decreased cell infiltration in the bronchi, pulmonary vessels, and neutral mucus production. Acetate was administered 15 days prior to sensitization and continued until one day before the first challenge (day 20).Ten days after the last challenge, the ovaries were removed. Ten days after ovariectomy (OVx), animals underwent rechallenge. All parameters were assessed 24 hours after the last rechallenge. **(A)** Representative hematoxylin and eosin (H&E) staining, and periodic acid–Schiff (PAS) staining of lung tissue from the OVx, OVA-OVx-PBS, and OVA-OVx-acetate groups. Scale bars = 200 μm. **(B)** Quantification of peribronchial, **(C)** perivascular infiltration scores, and **(D)** neutral mucus. All the results represent data from two different experiments and are expressed as the means ± SEMs (n = 6-7 in all the groups). Statistical significance was determined by one-way analysis of variance (ANOVA), followed by Tukey’s *post hoc* test. *P < 0.05, **P < 0.01, ****P < 0.0001.

**Figure 3:**
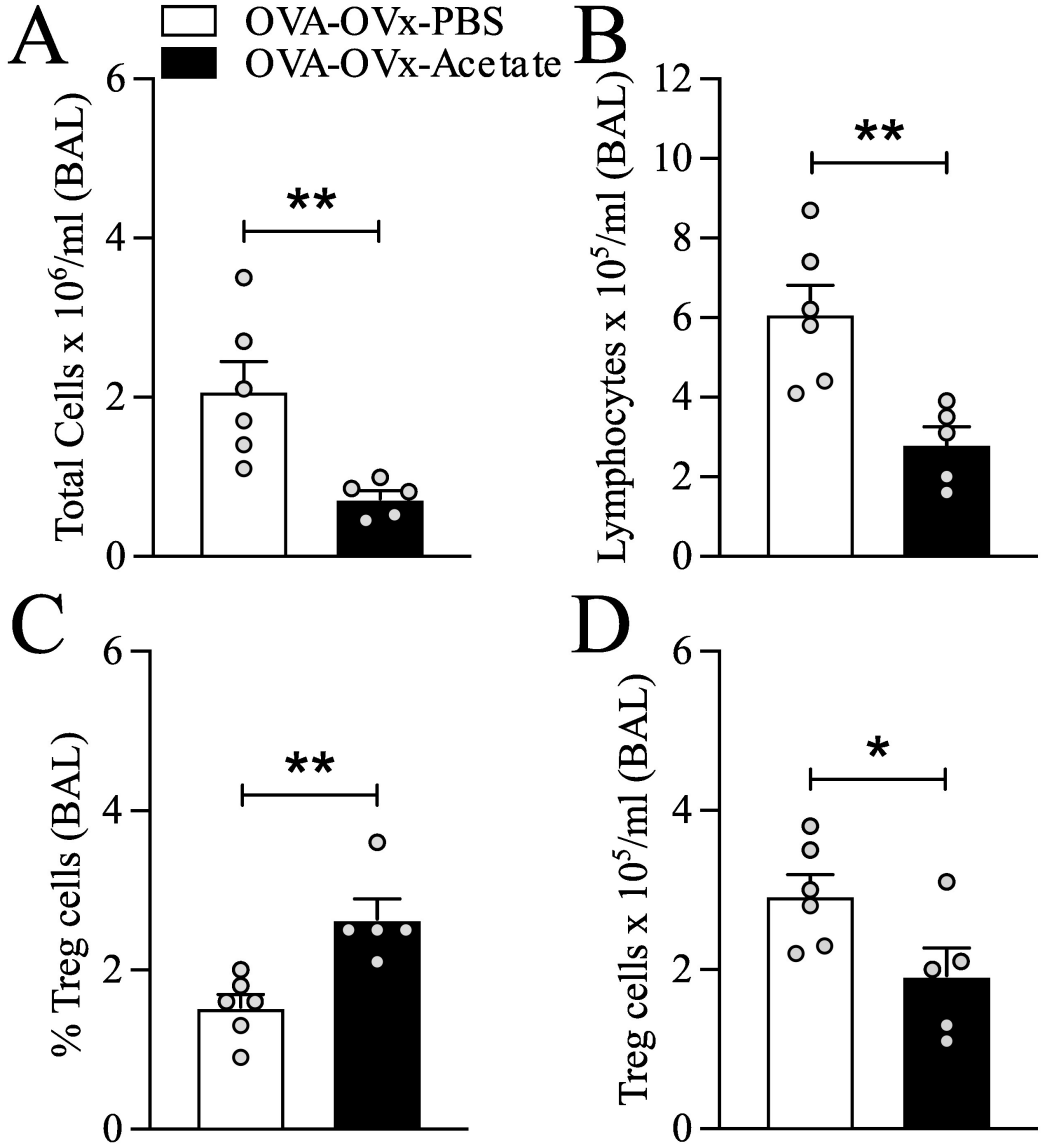
Preventive acetate treatment enhances the percentage of lung Treg cells in re-challenged OVx allergic mice. Acetate was administered 15 days prior to sensitization and continued until one day before the first challenge (day 20).Ten days after the last challenge, the ovaries were removed. Ten days after ovariectomy (OVx), animals underwent rechallenge. Twenty-four hours after rechallenge, cells from the BAL fluid were analyzed by flow cytometry and stained for CD4-APC, CD25-PE, and FoxP3-FITC. **(A)** Total cells, **(B)** Lymphocytes, **(C)** Percentage of Treg cells and **(D)** Number of Treg cells. All the results are expressed as the means ± SEMs (n = 6-7 in all the groups). Statistical significance was determined by one-way analysis of variance (ANOVA), followed by Tukey’s *post hoc* test. *P < 0.05, **P < 0.01.

Acetate was administered for 15 days prior to sensitization and continued until one day before the first challenge (day 20). From days 21 to 24, the animals were challenged with OVA. Ten days after the last challenge, they underwent ovariectomy, and ten days after ovariectomy (days 44 to 48), they underwent a rechallenge cycle. Euthanasia was performed 24 hours after the last rechallenge, followed by the collection of biological samples. This experimental protocol refers to **Figures 1**–**3**, **5**.

The female mice were distributed into two or three groups, depending on the experiment: OVx (n=6, ovariectomized mice that were not sensitized, challenged, or rechallenged with OVA), OVA-OVx-PBS (n=7, mice treated with PBS, challenged, and rechallenged with OVA), and OVA-OVx-acetate (n=7, mice treated with acetate, challenged, and rechallenged with OVA). The OVx group is shown in **Figures 2**, **5**, where histological analysis was performed.

**Figure 4:**
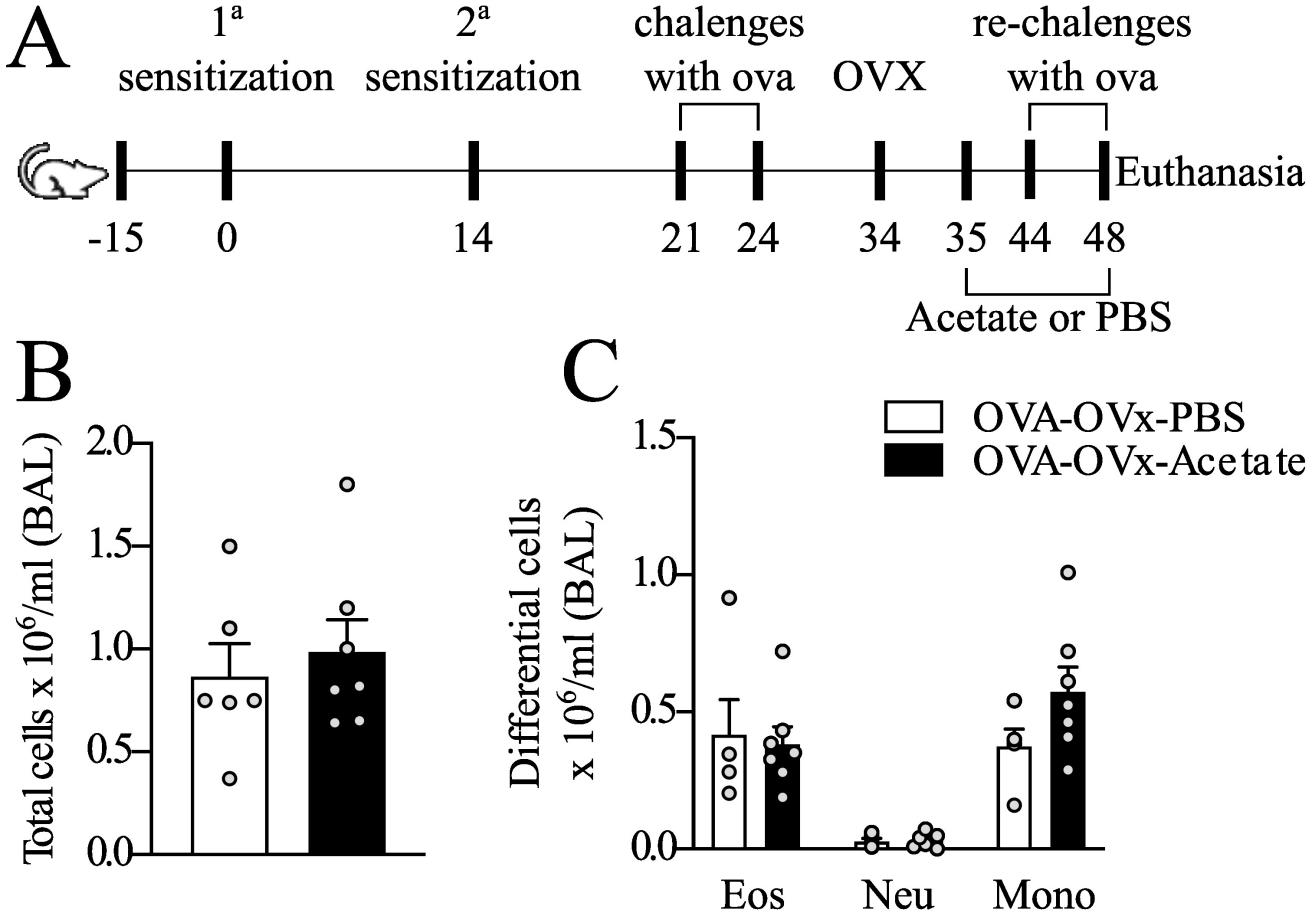
Effect of therapeutic acetate treatment on the number of cells recovered from BAL fluid in rechallenge OVx allergic mice. **(A)** Mice were sensitized and challenged. Ten days after the last challenge, the ovaries were removed, and the mice were treated with saline or acetate starting on Day 35, with treatments continuing every other day until Day 48. Ten days after the ovariectomy (OVx), the animals underwent rechallenge **(B)** Total cells count quantification **(C)** Quantification of eosinophils, neutrophils, and mononuclear cells. All the results represent data from two independent experiments and are expressed as mean the means ± SEMs (n =5–6 in all the groups). There were no significant differences among the groups investigated.

In the second experimental protocol, aimed at evaluating the therapeutic effect of acetate, asthma was induced, and the animals underwent ovariectomy as described earlier. However, starting one day after ovariectomy, the animals received intraperitoneal acetate treatment until the final challenge. This experimental protocol refers to **Figure 4**. The female mice were distributed into two groups: OVA-OVx-PBS (n=5) and OVA-OVx-acetate (n=6).

**Figure 5:**
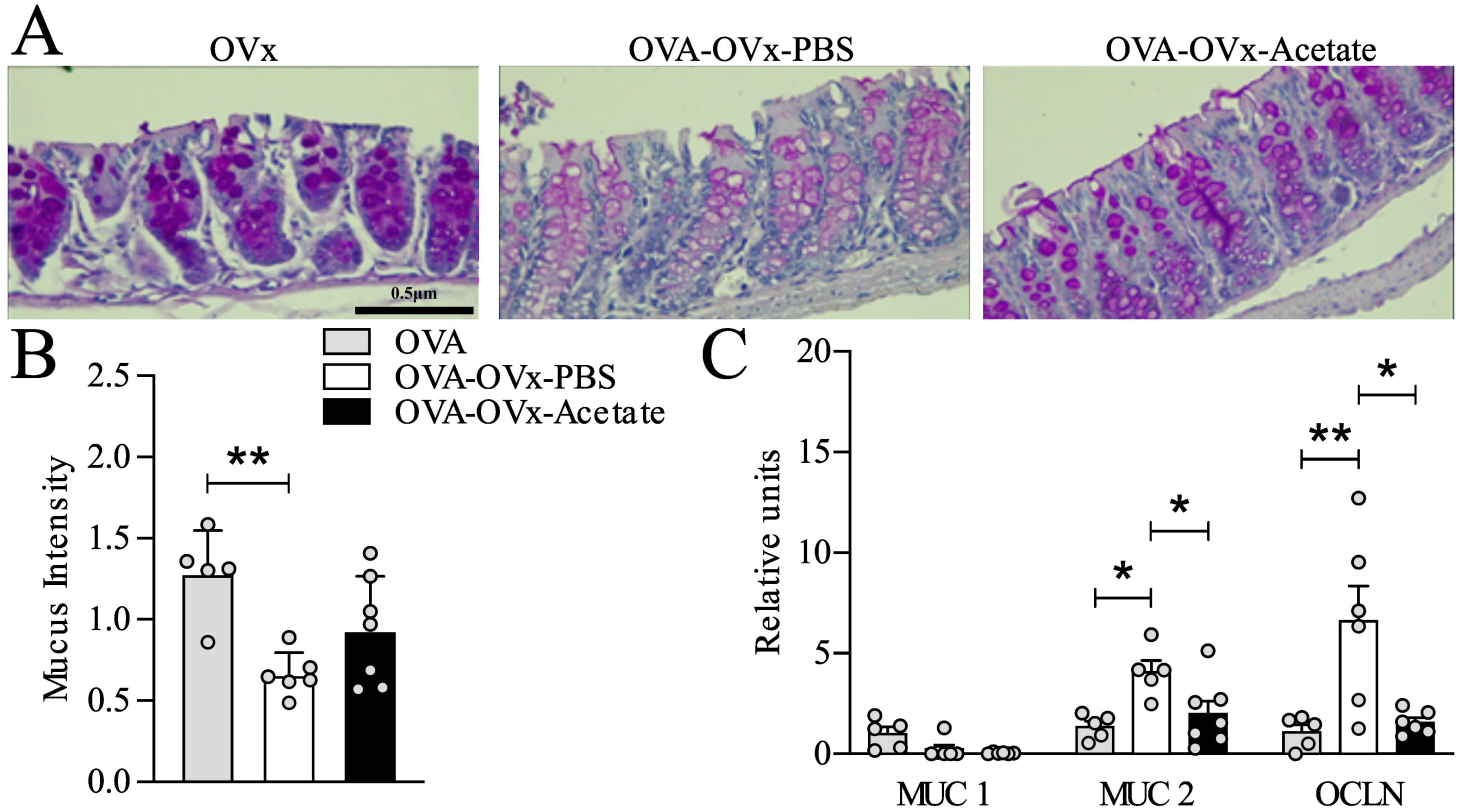
Effect of preventive acetate supplementation on mucus production and gene expression in the colon of rechallenge OVx allergic mice. Acetate was administered 15 days prior to sensitization and continued until one day before the first challenge (day 20). Ten days after the last challenge, the ovaries were removed. Ten days after ovariectomy (OVx), animals underwent rechallenge. All parameters were assessed 24 hours after the last rechallenge. **(A)** Representative periodic acid–Schiff (PAS) staining of distal colon. **(B)** Mucus intensity. **(C)** Relative mRNA expression levels of MUC1, MUC2, and OCLN were assessed by RT-qPCR in colon tissue from mice euthanized 24 hours after the last re-challenge. All the results represent data obtained from two separate experiments and are presented as the meanmeans ± SEMsmeans ± SEM (n = 5–6 in all the groups). Statistical significance was assessed using via one-way analysis of variance (ANOVA), followed by Tukey’s *post hoc* test. Values of *P<0.05 and **P<0.01 were considered statistically significant.

### 2.2 Animals

Female BALB/c mice (20–25 g) were obtained from the animal facility of the Federal University of São Paulo. The animals were housed in groups of five per cage in a light- and temperature-controlled room (12-hour light/dark cycles, 21 ± 2°C) with free access to food and water. The local Animal Care Committee of the Federal University of São Paulo approved the experiments (N˚1387060820). The female mice were separated into two groups: OVA-OVx-PBS and OVA-OVx-acetate.

### 2.3 Ovariectomy

Experimental OVx was performed as previously described with minor modifications (Mendes et al., 2021). The mice were anesthetized with 2% isoflurane and aseptically prepared with 2% chlorhexidine. An incision was made in the lower abdomen to access and surgically remove the ovaries from the surrounding tissue. Analgesic treatment included the subcutaneous administration of tramadol solution (Pfizer, 5 mg/kg) given 30 min before anesthesia induction and continued for three consecutive days. Additionally, carprofen (Rimadyl, 5 mg/kg s.c.) was administered once daily for three days postsurgery to manage postoperative pain. OVx efficacy was assessed by analyzing vaginal smear cell morphology and measuring uterine weights. The uterine tissues were removed immediately after euthanasia and weighed using a high-precision analytical balance. Any additional tissues were carefully removed before weighing to ensure accuracy Sham surgeries followed the same procedure but left intact ovaries in place. Ten days after OVx, we measured the body and uterus weights of the female mice and analyzed their vaginal morphology (**Table 1**). The OVx mice did not show a significant increase in body weight, but they presented significantly lower uterine weights than did the animals that underwent sham surgery (sham group). Furthermore, from a morphological perspective, the presence of vaginal cells characteristic of the diestrus phase of the estrous cycle was observed.

**Table 1:** Animal Weights and Ovariectomy Status.

Group	Number mice	Body weight (g)	Body mass gain (g)	Uterus weight (g)	Diestrus phase
Sham	5	19.91 ± 0.73	1.86 ± 0.31	66.00 ± 17.28	Negative
OVx	5	19.38 ± 1.76	2.12 ± 0.32	21.20 ± 5.63 **	Positive
OVA-OVx-PBS	6	22.40 ± 1.38	3.01 ± 1.33	16.50 ± 5.35 **	Positive
OVA-OVx-Acetate	6	22.91 ± 1.99	4.24 ± 2.75	16.67 ± 3.50 ****	Positive

The data were analyzed via one-way ANOVA and are presented as median values with ranges for body weight, body mass gain, uterus weight, and diestrus phase. Statistical significance is indicated by **p<0.01 and ****p<0.0001 compared with the sham group.

### 2.4 Treatment with acetate

The mice were 7 weeks old when the sodium acetate treatments began (Sigma−;Aldrich, St. Louis, MO, USA). The treatment involved the intraperitoneal (i.p.) administration of an acetate solution at a dosage of 1 g per kg body weight dissolved in distilled water, The pH of the solution was adjusted to 7.4 using sterile NaOH or HCl to ensure physiological compatibility. Acetate or saline (control group) administration started 15 days before exposure OVA and continued every other day until one day before the first challenge. Therapeutic treatment with acetate or saline was performed one day after OVx.

### 2.5 Induction of allergic lung inflammation

The mice were sensitized via i.p. injection of 30 µg OVA (OVA grade V, Sigma Chemical Co., Saint Louis, MO, USA) dissolved in 200 µL sterile PBS and 2 mg alum (Prod#77161, Thermo Scientific, Rockford, USA) on days 0 and 14. On days 21, 22, 23 and 24, the mice were challenged with aerosolized OVA (1% in PBS) for 30 min via an ultrasonic nebulizer device (Pulmosonic Star^®^, SP, Brazil) in a plastic inhalation chamber (18.5 cm X 18.5 cm X 13.5 cm), at a concentration of 0.1 g/10 mL. Ten days after the last challenge, the animals were subjected to OVx, and 10 days later, they were rechallenged for 4 days as described above. All assessments of inflammatory markers were conducted 24 h after the conclusion of the aerosol rechallenge phase (**Figure 1**).

### 2.6 Bronchoalveolar lavage fluid

Female mice were anesthetized with ketamine/xylazine (100 and 20 mg/kg, respectively). The trachea was exposed, and a cannula was inserted for BAL fluid collection. BAL fluid was obtained by washing the lungs twice with 0.8 mL of sterile cold saline injected through the cannula, which was repeated twice for a total volume of approximately 1.6 mL. The BAL fluid was then centrifuged, and the supernatant was used for cytokine analysis. Total cell counts were determined via a Countess^®^ automated cell counter (Invitrogen, Carlsbad, CA, USA). Differential cell counts were performed via cytospin analysis, and aliquots of BAL fluid (200 µL) were prepared via centrifugation at 300 × g for 1 min via a cytocentrifuge (Fanem Cytocentrifuge, SP, Brazil). The cells were stained with Instant Prov (Newprov, SP, Brazil), and a total of 300 cells were counted to determine the proportions of neutrophils, eosinophils, and mononuclear cells on the basis of standard morphological criteria.

### 2.7 Measurement of cytokine production

BAL fluid samples from animals were collected and centrifuged at 1200 rpm for 5 min. For lung tissue sample preparation, 1 mL RIPA lysis buffer (composed of 50 mM Tris-HCl at pH 7.4, 150 mM NaCl, 1 mM EDTA, 1% NP-40, and 0.25% Na-deoxycholate) supplemented with protease inhibitors (100 mg tissue per milliliter buffer) was used for sample processing. After homogenization, the samples were centrifuged at 8000 rpm for 10 min. The BAL fluid and lung tissue supernatants were stored at -80°C for cytokine analysis. For the BAL, the concentrations of cytokines interleukins (ILs) 4, 5, 13, 10, 17A and 33; INF-γ; and TNF-α were assayed via multiplex assays according to the manufacturer’s instructions for Mouse Magnetic Luminex^®^. Lung tissue samples were processed using the ELISA technique, employing specific kits from R&D Systems for the following cytokines: IL-4 (code DY404-05), IL-5 (code DY405-05), and IL-13 (code DY413-05). All the measurements were conducted in duplicate.

### 2.8 Measurement of immunoglobulins in serum

To quantify specific IgG1 and IgG2a immunoglobulins targeting OVA, ELISA plates were coated overnight at room temperature with an OVA solution (100 µg/mL) in coating buffer. The next day, the plates were washed three times with wash buffer (PBS containing 0.05% Tween-20) and then blocked for 1 h with assay diluent (PBS with 10% fetal bovine serum). Mouse serum samples diluted appropriately (1:500) in test diluent were added to the plates after three additional washes. After 2 h of incubation at room temperature, the plates were washed five times and incubated with anti-IgG1 and anti-IgG2a antibodies for 1 h. Following another wash, substrate reagent was added, and the plates were incubated in the dark for 30 min. The colorimetric reaction was stopped with a 2 N sulfuric acid solution. The absorbance was measured at 450 nm via a Biotek Synergy HT microplate reader. Sample values were determined by subtracting the absorbances of wells containing only fresh mouse serum at the same dilutions. The results are expressed in optical density (OD) units. ELISAs were conducted to analyze IgE levels in the serum via the BD Bioscience method following the manufacturer’s instructions.

### 2.9 Flow cytometry

Mouse Treg cells were collected from the BAL fluid and analyzed for CD4/CD25/Foxp3 expression via a mouse Treg cell staining kit (eBioscience). Briefly, the cells (1 × 106) were washed via centrifugation with cold PBS, resuspended in 1 ml of fixation/permeabilization solution, and incubated in the dark at 4 °C for 30–60 min. After being washed with permeabilization buffer, the cells were incubated with a blocking agent containing 2% normal rat serum in permeabilization buffer at 4 °C for 15 min. Fluorochrome-conjugated antibodies or isotype controls in permeabilization buffer were added, and the mixture was incubated in the dark at 4 °C for 30 min. After being washed, the cells were resuspended in flow cytometry buffer (PBS with 2% FBS) and analyzed via a FACSCanto II cytometer and FACS Symphony A1(BD Bioscience, San Diego, CA, USA). FlowJo^®^ software was used for data analysis.

### 2.10 Analysis of histological changes to lung and gut tissue

After the BAL fluid was collected, the left lungs were removed from the mice and fixed by immersion in 10% paraformaldehyde. The lobes were sagittally sectioned, embedded in paraffin, and cut into 5-µm sections for periodic acid-Schiff (PAS) staining. PAS staining was used to quantify the amount of neutral mucus in the lung tissue samples, involving measurement of the mucus area relative to the epithelial area. Image-Pro Plus 4.5 software facilitated this analysis. Each animal in each group had five bronchial airways examined, and images were captured at 40X magnification. Inflammatory rings in the airways were quantified via hematoxylin & eosin (HE) staining, with peribronchial and perivascular scores assigned as follows: 0 for normal conditions, 1 for slight inflammatory cell presence, 2 for a one-cell-deep ring, 3 for a two- to four-cell-deep ring, and 4 for a ring more than four cells deep (Tong et al., 2006). Each animal in each group had five bronchial airways and five pulmonary vessels analyzed, with images of the bronchial airways taken at 40X magnification. To quantify neutral mucus in the distal colon samples, we employed PAS (Periodic Acid-Schiff) staining. A portion of the distal colon was removed immediately after euthanasia and fixed in 10% buffered formaldehyde (Synth^®^, Diadema, São Paulo, Brazil). After a minimum fixation time (48 h), this tissue was subjected to dehydration, xylol clearing, bathing and paraffin embedding. Then, 3-μm sections were stained with periodic acid-Schiff (PAS). The analysis utilized Image-Pro Plus 4.5 software to quantify 10 photographs per animal for each group. Results were obtained by calculating the ratio of dark staining intensity to light staining intensity.

### 2.11 Measurement of MUC 1, MUC 2 and OCLN expression levels

Gene expression levels related to tissue homeostasis in the distal colon were assessed via real-time polymerase chain reaction (PCR). Total RNA was extracted using TRIzol™ Reagent (Invitrogen Life Technologies) and reverse transcribed into cDNA via an Applied Biosystems High-Capacity cDNA Kit. Real-time RT−;PCR was performed using StepOne™ Real-Time PCR equipment (Thermo Fisher) and SYBR Green fluorescent dye (Platinum^®^ SYBR^®^ Green qPCR Supermix UDG, Invitrogen). The sequences of primers used for amplifying the MUC 1 gene were as follows: forward primer: 5’-TCACCCCAGTTGTCTGTTGG-3’ and reverse primer: 5’-TCCTCATAGGGGCTACGCTT-3’. For the MUC 2 gene, the primers used were as follows: forward primer: 5’-CAACTGAATCCTCGACGCCT-3’ and reverse primer: 5’-GGGAGGGGGAAGGAGTGGA TT-3’. For the OCLN gene, the primers used were as follows: forward primer: 5’-GTGAATGGGTCACCGAGGG-3’ and reverse primer: 5’-AAGATAAGCGAACCTGCCGAG-3’. Gene expression analysis followed a method previously described by Liu and Saint (2002).

### 2.12 Statistical analysis

Statistical analysis was performed using Student’s t-test and one-way analysis of variance (ANOVA), followed by Tukey’s *post hoc* test in GraphPad Prism 8 (GraphPad Software, USA). The results are presented as the means ± standard errors of the mean (SEM). Significance levels are denoted as *p<0.05, **p<0.01, ***p<0.001, and ****p<0.0001.

## 3 Results

### 3.1 Preventive acetate supplementation attenuates exacerbated allergic airway inflammation in rechallenged allergic OVx mice

Previously, we demonstrated that re-exposure of allergic OVx mice to the antigen (OVA) exacerbates lung inflammation (Mendes et al., 2017; Mendes et al., 2021). In this study, we repeated the model and found that rechallenged OVx allergic mice (OVx/OVA) exhibited increased eosinophil numbers compared to Sham/OVA allergic mice. This confirms our previous findings that ovariectomy exacerbates preexisting allergic airway inflammation. (**Supplementary Table S1**).

To determine whether probiotics could protect from the increased exacerbation in OVx mice, we investigated the preventive effect of acetate supplementation (**Figure 1A**). Acetate supplementation significantly reduced the total number of total cells and eosinophils recruited to the BAL fluid (**Figures 1B, C**). There was no difference in the numbers of mononuclear cells and neutrophils between the groups (**Figures 1D, E**). Additionally, there was a significant decrease in the interleukin-5 (IL-5), IL-10, and IL-13 levels in BAL fluid (**Figure 1G**), as well as a reduction in the IL-5 levels in lung tissue (**Figure 1F**). Furthermore, a significant decrease in total serum IgE levels was observed in allergic OVx mice that were rechallenged and had been treated with acetate during sensitization. (**Figure 1H**), but no change in IgG isotypes (**Figure 1I**). No significant differences were detected in IL-4, IL-17, IL-33, IFN-γ, IFN-α in the BAL fluid between groups (**Supplementary Figure 1**). Thus, acetate supplementation can specifically inhibit later allergic challenges after induced menopause.

### 3.2 Preventive acetate supplementation reduces subsequent mucus production in the airways of rechallenged allergic OVx mice

We assessed the impact of preventive acetate treatment on peribronchial and perivascular cell infiltration as well as mucus presence in the pulmonary airways (**Figure 2**). Our findings indicated that allergic ovariectomized mice treated with acetate preventively exhibited a significant reduction in peribronchial and perivascular cell infiltration (**Figures 2C, D**), as well as a decrease in mucus presence in the airways compared to the Sham allergic group (**Figure 2E**).

### 3.3 Preventive acetate supplementation increases the percentage of Treg cells in rechallenged allergic OVx mice

Preventive supplementation with *B. longum* 5^1A^ increases the production of SCFAs and the percentage of lung Treg cells in rechallenged allergic OVx mice (Mendes et al., 2017). We aimed to determine the impact of acetate treatment on Treg cells by analyzing Treg cells in BAL fluid using flow cytometry. Compared to untreated mice, rechallenged allergic OVx mice supplemented with acetate showed a significantly increased percentage of Tregs in BAL fluid (**Figure 3C**). However, the overall number of Tregs in BAL (**Figure 3D**) fluid decreased due to a reduction in total cells and lymphocytes in BAL in rechallenged allergic OVx mice (**Figures 3A, B**).

### 3.4 Acetate supplementation after OVx does not attenuate exacerbated lung inflammation

Next, we investigated the response to therapeutic acetate treatment in BAL fluid following OVx. Allergic mice were supplemented with acetate post-OVx until the final rechallenge day. However, our findings revealed no significant differences in the total and differential cell counts between the groups in this context (**Figures 4A–C**).

### 3.5 Preventive acetate supplementation affects gut Gene expression and mucus production in rechallenged allergic OVx mice

Our previous data from an OVA experimental model suggested that respiratory system challenges might not only lead to allergic airway inflammation but also disrupt gut homeostasis (Nascimento et al., 2023). To determine whether OVx combined with allergic lung inflammation affects the expression of specific genes and whether preventive acetate supplementation could reverse these effects, we analyzed mucus production and gene expression in the distal colon. Mucus production was assessed using PAS staining, given the differing gene expression levels in the distal region. We observed a decrease in PAS intensity in allergic OVx mice treated with PBS compared to the non-allergic OVx group (**Figures 5A, B**). In the acetate-treated group, four out of seven animals showed increased mucus intensity; however, this difference was not statistically significant (**Figure 5B**). We also analyzed the gene expression of MUC1, MUC2, and OCLN in distal colon samples. Compared to nonsensitized OVx mice, rechallenged allergic OVx mice exhibited decreased gene expression of MUC1, which preventive acetate treatment failed to reverse (**Figure 5C**). Conversely, allergic OVx mice showed increased gene expression of MUC2 and OCLN. Remarkably, preventive administration of acetate successfully reduced the expression of these two genes (**Figure 5C**).

## 4 Discussion

Menopause is often linked to the onset or worsening of preexisting asthma. While late-onset asthma during menopause is associated with a Th17 profile, it is the exacerbation of asthma during this period that is particularly characterized by a T2 profile. This T2-driven asthma during menopause can manifest with increased eosinophilic inflammation and heightened sensitivity to allergens (Xie et al., 2022). In this study, we contribute to the understanding of T2 asthma, which is an existing allergic asthma that worsens as a woman enters menopause. Here, we investigated the effects of acetate treatment on the exacerbation of allergic lung inflammation in OVx mice. By focusing on the effects of acetate supplementation, our research aims to uncover potential therapeutic benefits for managing menopausal asthma exacerbations.

Ovariectomy exacerbates pulmonary inflammation in allergic animals. Here, we evaluated the impact of preventive acetate treatment on various inflammatory parameters in the airways of allergic ovariectomized mice. Our results indicated that preventive acetate treatment reduced levels of several inflammatory markers, including eosinophilic infiltration and the production of IL-5, IL-13, and IL-10 in BAL fluid, as well as the production of IL-33, IL-5, and IL-13 in lung tissue homogenates. Additionally, we observed reductions in serum IgE levels, mucus production, and lung tissue inflammation. These findings are consistent with our previous studies, which showed that allergic OVx animals exhibited exacerbated allergic asthma and that prophylactic treatment with the acetate-producing probiotic *Bifidobacterium longum* 5^1A^ alleviated the aggravation of allergic lung inflammation (Mendes et al., 2017).

The exogenous administration of acetate has been extensively studied (Kimura et al., 2011; Trompette et al., 2014; Ciarlo et al., 2016). One study examined the levels of exogenous acetate administered to mice and reported a significant increase in plasma acetate levels in both males and females 15 minutes after intraperitoneal (i.p.) injection (Ciarlo et al., 2016). Although we did not measure acetate concentrations in plasma in this study, several studies in the literature have assessed basal acetate levels, as well as those following intraperitoneal injections or a high-fiber diet (Kimura et al., 2011; Trompette et al., 2014; Ciarlo et al., 2016; Shubitowski et al., 2019). Administering acetate i.p. in mice allowed us to exclude the direct effects of acetate on the intestine and to understand its systemic effects. However, it would also be beneficial to explore the administration of acetate through drinking water or a fiber-rich diet. These methods might provide insights more akin to intragastric administration of probiotics, which also promote an increase in intestinal acetate and, subsequently, its presence in the circulation.

Another important aspect is that acetate treatment was administered at the same site where the animals were sensitized with OVA/Alum. This raises the possibility that the observed effects result from interference with OVA trafficking and presentation in draining lymph nodes. However, the systemic effects of acetate should also be considered, as previous studies have shown that even when sensitization occurs via the respiratory tract (e.g., with house dust mite), intraperitoneally injected acetate can still suppress airway eosinophilia (Trompette et al., 2014). Moreover, our findings suggest that acetate does not interfere with antigen presentation in draining lymph nodes, as indicated by the unchanged levels of OVA-specific IgG1 and IgG2a, which reflect intact systemic Th2 priming. However, the significant reduction in IL-5 and IL-13, along with an increase in regulatory T cells, suggests that acetate primarily modulates the effector phase of the allergic response likely through mechanisms acting at the pulmonary level rather than during the initial sensitization stage. Although this evidence provides valuable insights, further research is required to gain a deeper understanding of this aspect in the future.

GPR43 serves as the principal receptor for acetate and is present in many immune cells. Notably, when compared to wild-type mice, GPR43-deficient mice exhibit greater lung inflammation induced by the OVA protocol. This confirms that the activation of GPR43 by SCFAs is crucial for attenuating lung inflammation (Maslowski et al., 2009). Given this context, we examined whether the effects of acetate are mediated by Treg cells. Studies in animal models have demonstrated that Tregs are able to inhibit T2 pathway inflammation in airways, increasing tolerance to allergens (Gracias et al., 2019; Gracias et al., 2021; Zhang et al., 2022). We observed a significant reduction in the percentage of Treg cells in allergic ovariectomized and rechallenged animals treated with acetate compared to those treated with saline. SCFAs can modulate the immune system through various mechanisms, including the activation of G protein-coupled receptors, such as GPR43, and the inhibition of histone deacetylases (HDAC) (Vinolo et al., 2011). HDAC inhibition is known to induce regulatory T cells (Tregs). Interestingly, HDAC9 plays a role in suppressing FoxP3 and IL-2 (Wang et al., 2015). Studies have demonstrated that a high-fiber diet increases SCFAs, particularly acetate, which suppresses HDAC9 and enhances Tregs during allergic pulmonary inflammation (Trompette et al., 2014; Thorburn et al., 2015; Du et al., 2024). In this study, we demonstrated that preventive acetate treatment during sensitization, prior to OVx, is associated with prolonged protection against the Th2 response, alongside an increase in the percentage of Treg cells. In addition, the increase in the percentage of Tregs in the allergic ovariectomized group treated with acetate was accompanied by a reduction in IL-10 levels. Future studies are needed to determine which cytokines these Tregs are expressing, such as TGF-β, as well as to identify the factors contributing to the decrease in IL-10 in the bronchoalveolar lavage. Additionally, further investigation is required to determine which cells are responsible for IL-10 production in our experimental model.

Furthermore, Tregs are involved in maintaining the balance of Th1/Th2 inflammation (Chapoval et al., 2010) and may be associated with a reduction in IL-5 levels in the BAL fluid of allergic OVx and rechallenged mice treated with acetate. IL-5 is a pivotal cytokine in the differentiation and maturation of eosinophils in the bone marrow and is considered an important factor for their survival, as eosinophil-related apoptosis is delayed in the presence of this cytokine (Theiler et al., 2019). Interestingly, research has indicated that SCFAs, including acetate, can modulate eosinophil migration and survival by binding to the GPR43 receptor (Maslowski et al., 2009; Verstegen et al., 2021). We also found reduced levels of IL-13 in the BAL fluid and lung tissues of allergic and OVx mice treated with acetate. IL-13 is a type 2 cytokine associated with lung epithelial cell hypertrophy and mucus production (Zhu et al., 1999; Fort et al., 2001; Kuperman et al., 2002; Jackson et al., 2020). Histological and morphometric analyses of the lungs, revealing a decrease in neutral mucus production and a reduction in the sizes of inflammatory cell rings around bronchi and vessels in the acetate-treated groups. Increased levels of neutral mucus are associated with worsening asthma (Cohn et al., 1997; Grünig et al., 1998; Alevy et al., 2012; Martínez-Rivera et al., 2018). Previous studies performed in our laboratory involving preventive treatment with acetate in A/J mice also revealed that acetate treatment is able to decrease lung mucus production (Casaro et al., 2021). In addition, in a mouse model of rhinoviruses, intranasal administration of acetate reduced Muc5ac expression (Antunes et al., 2023). Besides IL-5 and IL-13, we found decreased levels of IL-33 in the lung homogenate of mice treated with acetate. Interestingly, it was observed in intestinal cell cultures that IL-33 indirectly affects mucus production and MUC2 by regulating the production of IL-13 by innate lymphoid cells (Waddell et al., 2019). Given the similar functions of these cytokines in the lungs and gut, we can consider that the same might occur in the lung.

While acetate treatment was found to alleviate asthma exacerbation during menopause as a preventive measure, we further investigated whether acetate could be used as a treatment for managing existing exacerbations. We administered this treatment to allergic mice after OVx, but it was not capable of attenuating the exacerbated inflammatory response. Similar results have been reported in which treating allergic mice with an acetate-producing probiotic after OVx had no effect on the exacerbation of allergic airway inflammation (Mendes et al., 2017). These findings are important because they suggest that acetate treatment is effective only when the disease has not yet established itself in the lung. The main limitations of our study include the timing of treatment administration and the lack of investigation into long-term effects post-treatment. Thus, it would be beneficial to conduct further studies to investigate the effects of acetate after the first cycle of allergen challenge or prior to OVx. Our research group is currently studying the mechanisms involved in the preventive versus curative effects of acetate treatment, aiming to understand these dynamics better.

Given that this experimental model of asthma exacerbation in menopause is novel and little explored, especially in the context of the gut−;lung axis, we explored the expression of genes related to the colon barrier, such as MUC 1, MUC 2, and OCLN, in this novel model. We analyzed these genes 24 h after the last rechallenge; these genes are involved in the gut barrier or permeability (Chang et al., 2012; Grondin et al., 2020). An integral gut epithelial barrier has been shown to be relevant to health states since a barrier is responsible for the selectivity of access between the external environments (Barbara et al., 2021). Therefore, maintenance of the gut epithelial barrier is performed by a complex set of protein junctions and mucus (Barbara et al., 2021). Intestinal mucus from the colon plays an important role in intestinal lubrication and epithelial cells protection and is secreted by goblet cells and formed by a network of proteins, including MUC2, throughout the O-glycosylation process (Johansson et al., 2011; Rodríguez-Piñeiro et al., 2013; Van Der Post et al., 2019). MUC2 is synthesized by goblet cells in the intestinal epithelial layer and acts as the first barrier preventing direct contact between intestinal bacteria and colon epithelial cells. Among other functions, MUC2 expression plays an important role in the selection of the gut microbiota. The specific role of MUC2 expressed in the intestine in the context of asthma is not yet well established. A recent study from our group showed that animals with allergic lung inflammation exhibit increased MUC2 expression in the colon compared to animals that were sensitized with OVA but challenged with PBS, or those without pulmonary inflammation (Nascimento et al., 2023). These findings indicate that the intestinal changes are associated with pulmonary inflammation rather than sensitization alone. Here, we also found an increase in MUC2 expression in the rechallenged allergic OVx group compared with the baseline group. Interestingly, acetate treatment during sensitization is sufficient to restore the normal expression of MUC2 in the gut. Although MUC2 expression is increased in ovariectomized and allergic mice, the intensity of PAS staining is reduced. This may occur due to various factors: (a) altered glycosylation of MUC2, with fewer carbohydrates, (b) a defect in mucin secretion, (c) disorganized secretion and (d) accelerated degradation (Chang et al., 2012; Adler et al., 2013; Melhem et al., 2021). A study from our group, mentioned earlier, shows that pulmonary inflammation increases the expression of MUC2 but also decreases the intensity of PAS staining in the intestinal colon (Nascimento et al., 2023). Transmembrane mucins, such as MUC1, act at the surfaces of epithelial cells, forming a layer called the glycocalyx, which is a protective barrier against insults (Gipson, 2004). Compared with that in the basal group, the expression of MUC1 in allergic and OVx mice treated with acetate or not treated with acetate was lower, indicating that allergic and OVx mice may have an impaired gut barrier. Beside MUC1 and MUC2, we investigated the OCLN that is an important protein in the tight junctions of the colon (Nusrat et al., 2000). OCLN expression exhibited alterations similar to those of MUC2; the expression of OCLN in allergic and OVx mice was increased compared with that in the basal group, and acetate treatment restored basal expression. Regarding the effects of acetate on the intestine, we hypothesize that its impact may result from the reduction of pulmonary inflammation, which subsequently lowers cytokine and inflammatory mediator levels that could influence the intestine. Alternatively, intraperitoneally injected acetate may directly reach the intestine through absorption by mesenteric vessels or the lymphatic system. Further studies are needed to confirm these hypotheses. Clinical studies have demonstrated the association of increased OCLN gene expression with inflammatory bowel disease, such as ulcerative colitis (Yamamoto-Furusho et al., 2012). This is relevant since a certain percentage of asthmatic patients develop chronic intestinal diseases or vice versa. Additionally, gastrointestinal disorders, such as gastroesophageal reflux disease is indeed associated with changes in mucin expression, including MUC1 and MUC2 (Van Roon et al., 2008), and menopause is an important risk factor for gastroesophageal reflux disease and are related to the onset or worsening of asthma symptoms (Benard et al., 1996; Breugelmans et al., 2022; Freuer et al., 2022). A more thorough intestinal exploration is necessary, including an investigation of the gut microbiota and confirmation of gene expression changes through additional techniques.

**Figure 6:**
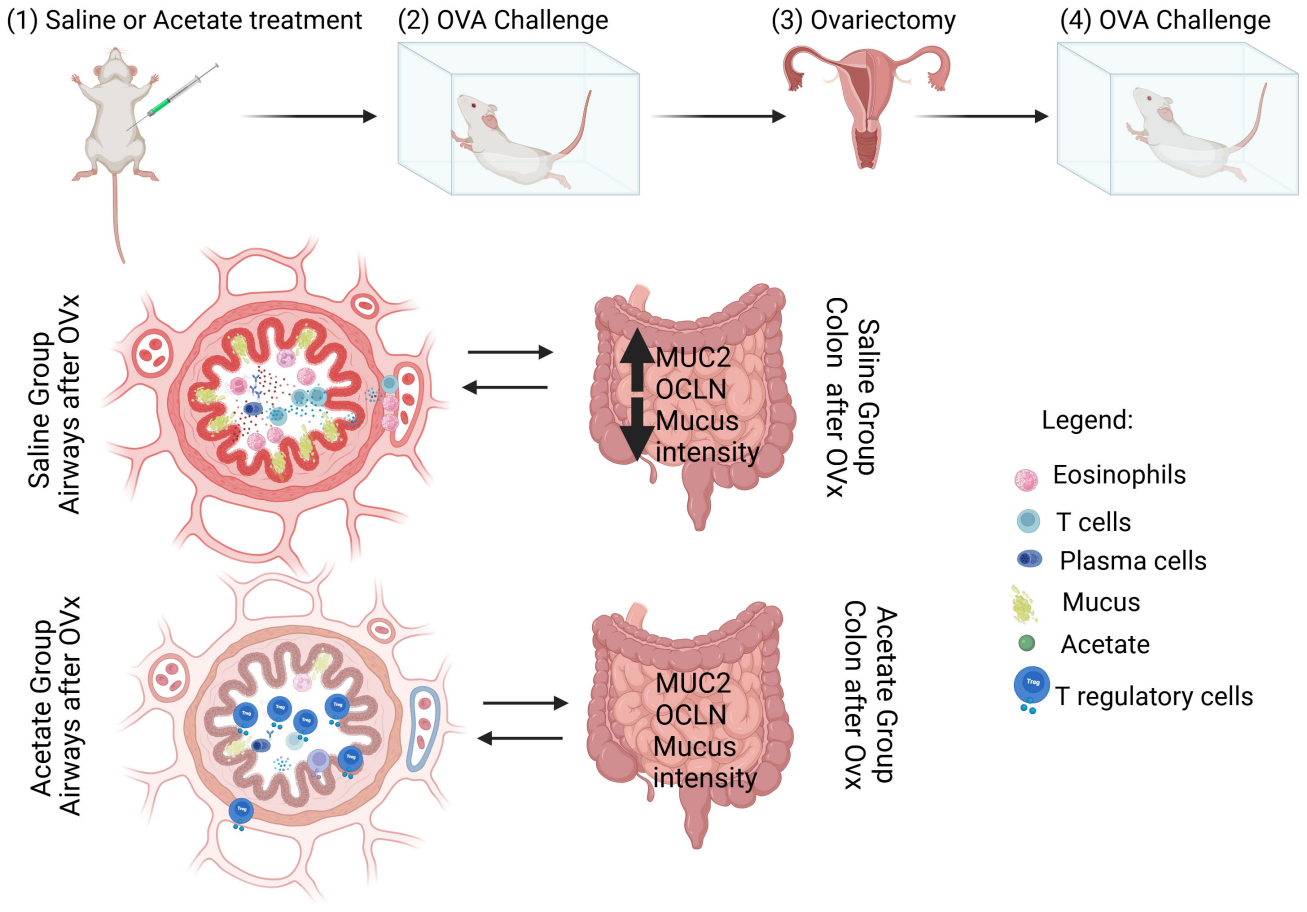
Schematic representation of the effect of acetate treatment on the lung and gut of rechallenged OVx allergic mice (Peacock et al., 2023). Acetate was administered 15 days prior to sensitization and continued until one day before the first challenge (day 20) (Colombo et al., 2019). Ten days after the last challenge, the ovaries were removed (Chowdhury et al., 2021). Ten days after ovariectomy (OVx), the animals underwent rechallenge. Twenty-four hours after rechallenge, cells from the BAL fluid and gut were analyzed. Mice treated with acetate showed a significant reduction in cell infiltration, mucus production, IgE, and cytokine levels, as well as an increased percentage of Treg cells in the airways compared to mice treated with saline. Additionally, acetate treatment increased the expression of MUC2 and OCLN and decreased mucus intensity in the gut. Created in BioRender. De oliveira ribeiro, F. (2025) https://BioRender.com/p79j710.

While few studies have addressed treatments for asthma exacerbation during menopause, the roles of microbial products in this context remain largely unexplored. This study is particularly impactful as it demonstrates that microbiota-derived acetate provides long-term protection against asthma exacerbation during menopause. This protective effect is achieved by reducing eosinophil cells, Th2 cytokines, and mucus production, with Treg cells playing a crucial role in these mechanisms (**Figure 6**). Additionally, acetate treatment influences gene expression in the gut during asthma exacerbation, which is especially relevant given that menopausal women often experience gut issues, such as gastroesophageal reflux disease, that are linked to asthma onset or exacerbation. Although further investigation is required to understand the optimal timing for acetate treatment during disease exacerbation, this study provides promising evidence of acetate’s protective effects. Notably, while administering acetate post-OVx did not alleviate airway inflammation in this study, the overall findings highlight the potential of acetate as a preventive measure and warrant further research to explore its therapeutic applications.

## Data Availability

The original contributions presented in the study are included in the article/**Supplementary Material**. Further inquiries can be directed to the corresponding author.
