# Supplementary material for: Exogenous acetate mitigates later enhanced allergic airway inflammation in a menopausal mouse model
Source: Front Cell Infect Microbiol. 2025 Apr 10;15:1543822. doi: 10.3389/fcimb.2025.1543822 (PMC12023485; doi:10.3389/fcimb.2025.1543822)
Supplement: Supplementary file 1 [file DataSheet1.pdf]

Supplementary Material

1 Supplementary Figures and Tables

1.1 Supplementary Figures

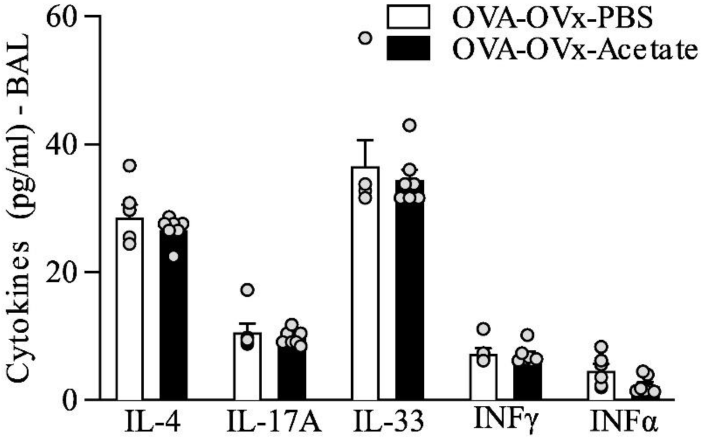

Supplementary Figure 1. Evaluation of cytokines in bronchoalveolar lavage. Acetate was administered 15 days prior to sensitization and continued until 20 days before the first challenge. Ten days after the last challenge, the ovaries were removed. Ten days after ovariectomy (OVx), the animals underwent rechallenge. Twenty-four hours after rechallenge, BAL was collected to quantify cytokines such as interleukin-4 (IL-4), interleukin-17A (IL-17A), interleukin-33 (IL-33), interferon-gamma (IFN-γ), and interferon-alpha (IFN-α). There were no significant differences among the groups investigated.

1.2 Supplementary Tables

Supplementary Table S1. BAL inflammatory cells in the nonsensitized and rechallenged Sham and OVx groups, as well as in the sensitized groups and sensitized and rechallenged Sham and OVx groups. The data were analyzed using one-way ANOVA and are presented as median values with ranges. Statistical significance is indicated as $^{\#\#}p < 0.01$ versus OVA-Sham, $^{***}p < 0.001$ versus Sham and OVx

**Bronchoalveolar lavage of recovered cells**			
**Group**	**Number of mice**	**Total cells in BAL ($\times 10^6$/mL)**	**Eosinophils ($\times 10^6$/mL)**
Sham	5	0.24 ± 0.119	0.00 ± 0.00
OVx	5	0.11 ± 0.023	0.00 ± 0.00
OVA-Sham	11	1.64 ± 0.734***	0.56 ± 0.23***
OVA-OVx	6	2.01 ± 0.435***	0.98 ± 0.27***;$^{\#\#}$
